# Enhanced salt tolerance in *Glycyrrhiza uralensis* Fisch. via *Bacillus subtilis* inoculation alters microbial community

**DOI:** 10.1128/spectrum.03812-23

**Published:** 2024-08-27

**Authors:** Jiancai Xiao, Jing Xiao, Pengchao Gao, You Zhang, Binbin Yan, Hongli Wu, Yan Zhang

**Affiliations:** 1 National Key Laboratory for Quality Ensurance and Sustainable Use of Dao-di Herbs, National Resource Center for Chinese Materia Medica, China Academy of Chinese Medical Sciences, Beijing, China; 2 Dongying Municipal Bureau of Agriculture and Rural Development, Shandong, China; 3 Laiwu City Ziguang Ecological Park Co, Shangdong, China; 4 Institute of Basic Research In Clinical Medicine, China Academy of Chinese Medical Sciences, Beijing, China; Fujian Agriculture and Forestry University, Fuzhou City, Fujian, China

**Keywords:** *Glycyrrhiza uralensis *Fisch, salt stress, *Bacillus subtilis*, microbiome, assembling process

## Abstract

The widespread prevalence of saline environments poses a significant global environmental challenge. Salt stress, induced by saline soils, disrupts soil microecology and affects the plant-microbe-soil cycling process. Utilizing microbial fungicides stands as a primary strategy to mitigate salt stress-induced damage to plants and soils. This study investigated the influence of *Bacillus subtilis* (Bs) inoculation on the microbial community, assembly processes, and functional changes in bacteria and fungi in *Glycyrrhiza uralensis* Fisch. (licorice) seedlings under varying salt stress levels, primarily employing microbiomics techniques. Soil enzyme activities displayed a declining trend with increasing salt stress, which was mitigated by Bs inoculation. Microbiome analysis revealed a significant increase in bacterial and fungal operational taxonomic units, particularly in Ascomycetes and Nitrogen-fixing Bacteria, thereby enhancing soil denitrification. The abundance of Proteobacteria, Actinobacteriota, Bacteroidota, and Firmicutes in bacteria, as well as Ascomycota in fungi, increased with higher salt stress levels, a process facilitated by Bs inoculation. However, functional predictions indicated a reduction in the relative abundance of Dung Saprotrophs with Bs inoculation. Salt stress disrupted soil assembly processes, showcasing a continuous decline in diffusion limitation with increased salt concentration, where Bs inoculation reached a peak under moderate stress. In summary, this research elucidates the communication mechanism of Bs in enhancing salt tolerance in licorice from a microbiome perspective, contributing to a comprehensive understanding of abiotic and biotic factors.

**IMPORTANCE:**

Licorice is a herb that grows in deserts or saline soils. Enhancing the salt tolerance of licorice is necessary to maintain the quality of cultivated licorice and to ensure the supply of medicinal herbs. In the past, we have demonstrated the effectiveness of inoculation with *Bacillus subtilis* (Bs) to enhance the salt tolerance of licorice and revealed the key metabolic pathways for the development of salt tolerance through multi-omics. In this study, we used the microbiomics approach to reveal the plant-microbe-soil interactions at the level of inoculation of Bs affecting the dynamics of soil microbial communities from bacterial and fungal perspectives, thus bridging the interactions between biotic and abiotic factors.

## INTRODUCTION

Salt-affected areas are characterized by high salt concentrations and elevated pH levels, posing a globally prevalent and challenging environmental issue, rendering millions of hectares unsuitable for crop cultivation (1, 2). Salt stress is a primary consequence of soil salinity, causing medicinal plants grown in saline soils to synthesize secondary metabolites (3). However, high salt concentrations adversely affect both plants and soil ecosystems. This stress not only induces osmotic stress in plants, resulting in root cell damage and membrane lipid peroxidation, but also brings significant changes in soil physicochemical properties, nutrient supply, microbial diversity, and microbial assemblage processes, ultimately compromising soil fertility and ecosystem integrity (4, 5). Thus, addressing the challenges posed by salt stress is of paramount importance. Inoculating plants with microbial agents presents a promising strategy to enhance plant resistance to salt stress and has been proven effective in various plant species. The symbiotic relationship between these microbes, soil, and plants plays a crucial role in this process. These microbes, including bacteria, fungi, and arbuscular mycorrhizal fungi, influence the physiological and biochemical functions of plants, mediating the plant-microbe-soil cycling (6). *Bacillus subtilis* (Bs) has emerged as a research hotspot in helping plants cope with abiotic stress due to its unique properties. Bs, a spore-forming bacterium, produces heat and desiccation-resistant spores, surviving in harsh environments, and promoting plant growth (7). It forms a biofilm in the rhizosphere or symbiotically associates with roots, generating stress-resistant proteins and enhancing plant responsiveness to abiotic stress by altering soil microenvironment conditions, a conclusion validated in crops such as wheat (8) and rice (9).

*Glycyrrhiza uralensis* Fisch. (licorice), is a typical drought-resistant plant well-suited for sandy, desert, and semi-desert environments, possessing drought and saline-alkali tolerance, with the main medicinal parts being the roots and rhizomes (10). It is widely used in traditional Chinese medicine, revered as the “nine herbs of ten directions” and the “national old man” (11). Its ability to grow in salt-stressed environments is a key mechanism for it to become an “authentic medicinal material.” In recent years, mechanisms facilitating moderate salt stress in promoting effective compound synthesis and accumulation in licorice and influencing medicinal quality have been gradually elucidated. However, licorice is not a halophyte and cannot survive in highly saline-alkali soils. Given the increasing demand for licorice, coupled with limited land resources, exacerbated global climate change, reduced precipitation, and the widespread prevalence of saline-alkali environments, it is imperative to enhance its adaptability to salt stress (12). This is crucial for stabilizing the quality and yield of medicinal herbs. Furthermore, saline-alkali areas also impact the ecological environment; thus, enhancing licorice’s salt tolerance holds significant value in reclaiming saline-alkali and coastal areas, restoring ecological balance, and preserving biodiversity. While the predominant method to address abiotic pressure involves using microbial agents, most studies on their effectiveness remain largely focused on physiological and biochemical aspects, with the relatively weak exploration of the communication mechanisms these agents establish in the soil (13). This study involved subjecting licorice seedlings to varying degrees of salt stress concentrations and observing the ability of Bs to resist salt stress. In nature, plants often experience multiple types of salt stress, such as sodium chloride (NaCl) and calcium chloride (CaCl_2_). To better simulate natural conditions, this experiment used NaCl + CaCl_2_ as the primary salt stress factor. Our previous research indicated that inoculating Bs can enhance photosynthesis in plants and improve licorice seedlings' salt tolerance by enhancing plant cell wall functions (14). This study primarily employed 16S rRNA and ITS sequencing techniques to elucidate the changes in soil microbial community diversity and assemblage processes induced by Bs in response to salt stress, further enhancing our understanding of the cooperative mechanisms between abiotic and biotic factors.

## MATERIALS AND METHODS

### Experimental design and sampling

The rhizosphere soil and bulk soils utilized in this research were obtained from a licorice pot experiment involving varying levels of salt stress. Initially, licorice seeds that had been pretreated were sown in individual pots filled with a mixture of 4 kg of culture soil, quartz sand, and nutrient soil in a 4:1 ratio. For the group treated with Bs, 1 g of Bs was combined with 5 kg of water and uniformly incorporated into the soil during the mixing process (Bs was supplied by Shandong Muyushi Biotechnology Co. Ltd. and had an effective viable bacterial count exceeding 50 billion/g). Subsequently, the seeds were cultivated under standard greenhouse conditions, with 1/2 Hoagland nutrient solution administered every 10 days, and additional application of the bacterial solution for the Bs group every 15 days. The salt stress treatment commenced approximately 50 days into the growth phase when the seedlings had developed 8–10 true leaves. In this experiment, salt stress was administered at three different levels: 100, 200, and 300 mmol/L NaCl + CaCl_2_. The control group received no NaCl + CaCl_2_. All the aforementioned solutions were prepared using 1/2 Hoagland nutrient solution.

Based on the Bs inoculum and salt concentration levels (low, medium, and high), eight groups were formed, denoted as NC0, NCL, NCM, NCH, Bs0, BsL, BsM, and BsH. To minimize the effects of salt leaching, treatments with salt concentrations exceeding 100 mmol/L involved daily watering. The salt concentration was incrementally increased by 100 mmol/L until it reached the desired level, which was designated as day 0 of the treatment. After four separate waterings, each spaced 6 days apart, inter-root and field soils subjected to both Bs treatment and untreated controls were simultaneously sampled on the specified dates. Three replicates were obtained for each group, and the samples were promptly frozen in liquid nitrogen before storage at −80°C.

### Determination of enzymatic activities of S-CAT, S-SC, and S-UC

Soil catalase (S-CAT), soil sucrase (S-SC), and soil urease (S-UE) contents in seedling root tissues or soil were assessed using designated kits following the protocol provided by Beijing Solarbio Science and Technology Co. Ltd (www.solarbio.com). Absorbance readings were taken at 240, 540, and 630 nm using a UV spectrophotometer to quantify the respective parameters (15, 16).

### Soil DNA extraction, PCR, and high-throughput sequencing

DNA extraction was carried out on 0.5 g of freshly collected inter-root soil using the PowerSoil DNA Isolation Kit (Mo Bio, Carlsbad, CA, USA). The V3-V4 region of the bacterial 16S rRNA gene was amplified using the primer pairs 341F (5′-CCTAYGGGRBGCASCAG-3′) and 806R (5′-GGACTACNNGGGGTATCTAAT-3′). Additionally, the ITS1 region of the fungal ITS rRNA gene was amplified using the primer pairs ITS5-1737F (5′-GGAAGTAAAAGTCGTAACAAGG-3′) and ITS2-2043R (5′-GCTGCGTTCTTCATCGATGC-3′). PCR amplification of microbial sequences was performed on the Novaseq 6000 (Illumina). Raw sequences were subjected to quality screening and the QIIME (v.1.7.0) quality control process to obtain high-quality clean reads. Sequences were then clustered into operational taxonomic units (OTUs) at 97% similarity level after discarding individual tags.

### Co-occurrence network and microbiome assembly analysis

The present study conducted a nonrandom co-occurrence network analysis using OTUs detectable in ≥0.1% of cases to delve into intricate relationships within soil bacterial and fungal communities. In this analysis, we employed Spearman’s rank correlation to establish statistically robust associations between OTUs, considering correlations with a magnitude (|*r*|) greater than 0.6 and false discovery rate-corrected *P* values less than 0.01 as significant. Visualization of the networks was achieved using the interactive platform Gephi v.0.9.5. To gauge the complexity of the bacterial and fungal networks, we calculated various network metrics, including the number of nodes, edges, average degree, average weighted degree, density, and modularity. Moreover, we generated 1,000 Erdős-Rényi random networks using the “igraph” R package, maintaining an equivalent number of nodes and edges as the actual network, with each edge assigned to each node with uniform probability. The specific parameter settings and procedures adhered to those established in prior published studies. To discern potential drivers of community assembly, we employed the null model framework. Taxonomic β-diversity matrices, namely β-nearest taxon index (βNTI) and Bray–Curtis-based Raup–Crick (RCbray), were utilized to identify variations in the phylogenetic and taxonomic diversity of rhizosphere bacterial and fungal communities (17).

In particular, |βNTI| values exceeding 2 indicated that either variable or homogeneous selection (i.e., deterministic processes) played a significant role in shaping community composition. Conversely, |βNTI| values less than 2 suggested that stochastic processes were primarily responsible for the turnover between community pairs. Furthermore, RCbray values greater than 0.95 and less than −0.95 signified the relative influence of dispersal limitation and homogeneous dispersal, respectively. When |RCbray| values fell below 0.95, it indicated that microbial communities were predominantly shaped by undominated processes, which often encompassed weak selection, weak dispersal, diversification, and/or drift (18).

### Statistical analysis

Data normality and homogeneity were assessed using Shapiro-Wilk and Levene’s tests. For non-normally distributed data, a natural logarithm transformation was applied. Statistical analyses were conducted using SPSS software (version 24.0). Results are presented as mean ± standard deviation. Before performing one-way analysis of variance (one-way ANOVA), all data were checked for normality. The Tukey test was utilized for data analysis. Statistical significance was determined at *P* ≤ 0.05. For bacterial and fungal communities, the Shannon index and Chao index (community α-diversity), principal coordination analysis (PCoA) (community β-diversity), and major phyla of rhizosphere group with a relative abundance >1.0% were selected to test the community structure. Analysis of similarities (ANOSIM) was performed with 999 permutations to evaluate differences in microbial β-diversity. Significant differences were identified using the linear discriminant analysis effect size (LEfSe) method. Mantel test analysis of microbiome with transcriptome and metabolome data using ggcorrplot’s R package. Graphics were created using Origin 2021 and Adobe Illustrator CC 2021 to ensure clarity and accuracy (19).

## RESULTS

### Enzymatic activity of S-CAT, S-SC, and S-UC

**Fig 1:**
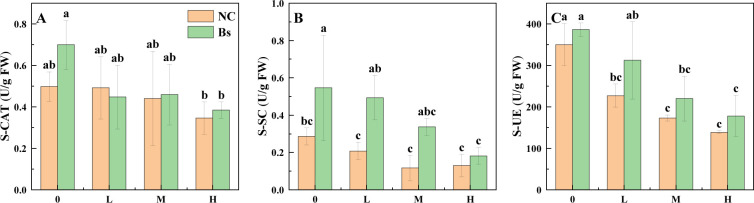
Analysis of S-CAT, S-SC, and S-UE at different salt concentrations (0, 100, 200, and 300 mmol/L). Data within the same followed by a string of the same lowercase letters are not significantly different (*P* > 0.05). At the same time, a string of different letters shows a significant difference (*P* < 0.05). Yellow represents the groups that were not inoculated with *Bacillus subtilis*, green represents the groups that were inoculated with *Bacillus subtilis*.

In this study, the effects of salt stress on soil enzyme activity were investigated. Our findings revealed that salt stress exerted varying degrees of inhibition on soil enzyme activity, with the inhibition positively correlated with the salt stress concentration (Fig. 1). However, inoculation with Bs increased the activities of three enzymes, S-CAT, S-SC, and S-UE, whether under salt stress conditions or not. Notably, the activity of S-SC showed the most significant improvement (Fig. 1B), increasing by 90.91%, 137.5%, 188.89%, and 40.00% compared to the NC group.

### Soil microbial community diversity

**Fig 2:**
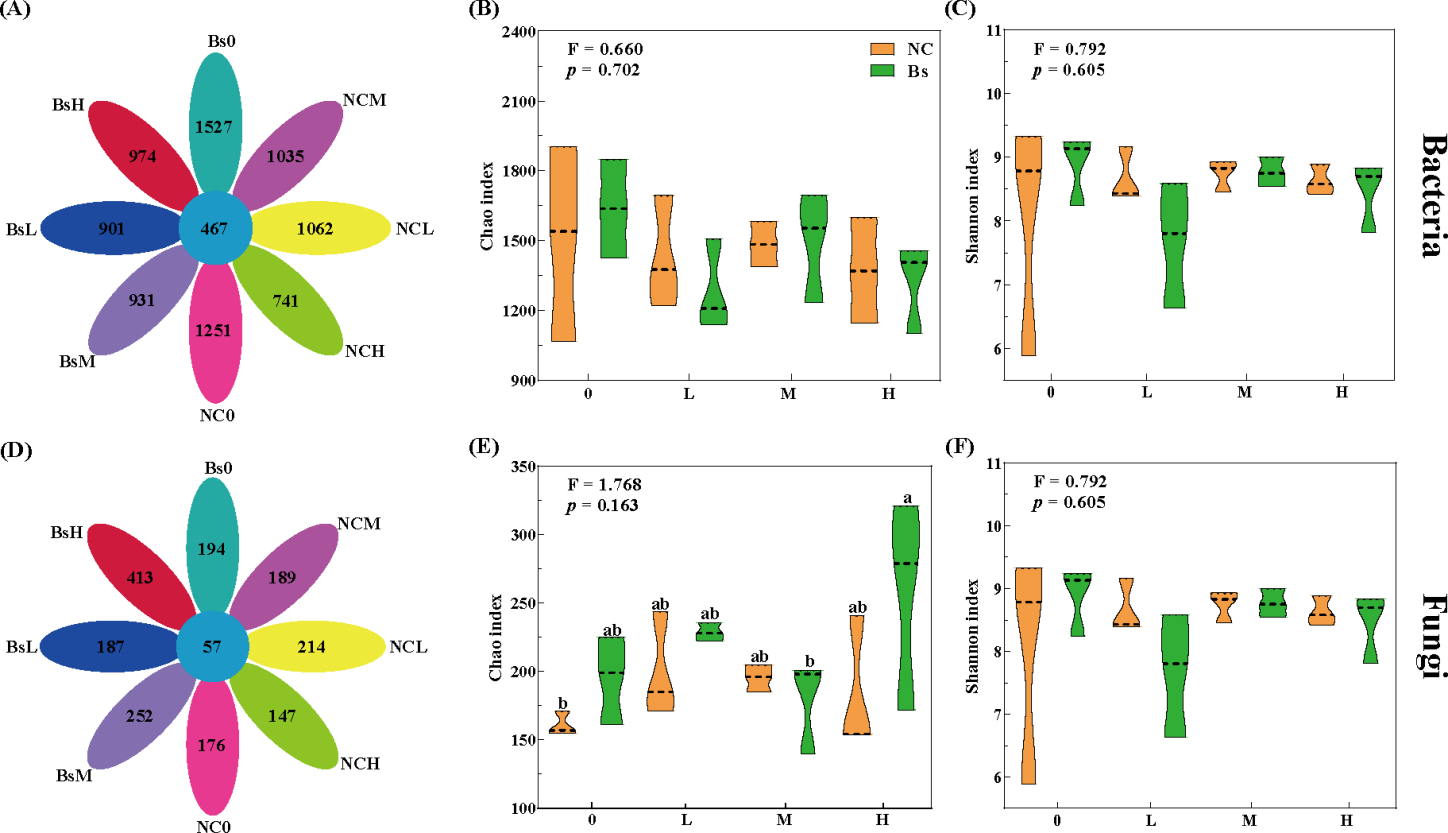
Alpha diversity of bacterial (**A–C**) and fungal (**D–F**) communities for the eight families. The petal plots depict the distribution of OTUs across various treatment groups, with the central core indicating the shared OTUs among the eight treatment groups. The Chao index and Shannon index denote species richness and diversity, respectively. The designations 0, L, M, and H correspond to low-medium and high-medium salt stress concentrations. The uninoculated Bs group is denoted by yellow, while the inoculated group is represented in green. Distinct lowercase letters within the same subplot denote statistically significant differences (*P* < 0.05).

After stringent quality control, the high-quality sequences of bacteria and fungi within all rhizospheric soil samples amounted to 1,907,832 and 1,818,851, respectively, with taxonomic assignments yielding 12,276 bacterial and 2,261 fungal OTUs at the species level. Illustrated through Venn diagrams, 467 bacterial OTUs, and 57 fungal OTUs were found to coexist between the eight treatment groups. Furthermore, within the same treatment group, the number of OTUs of bacteria was always higher than that of fungi, with bacteria having the most abundant OTUs values in the Bs0 group (1,527) and fungi in the BsH group (413) (Fig. 2A and D). Specifically, whether bacteria or fungi, the OTUs in the Bs group consistently exceeded those in the NC group, with the NCH group consistently demonstrating the lowest OTU count. Intriguingly, as salt stress escalated, the OTU count of bacteria or fungi inoculated from the Bs group exhibited a continuous rise, while the NC group displayed a consistent decline. Within both NC and Bs groups, bacterial α-diversity was highest without salt stress (including Chao index and Shannon index), peaking under moderate concentration post-stress and reaching its lowest under low concentration (Fig. 2B and C); concerning fungal α-diversity, salt stress heightened the Chao index, leaving the Shannon index largely unaffected. Remarkably, among these, the Chao index of the BsH group, upon Bs inoculation, significantly surpassed BsM and NC0 (*P* < 0.05) (Fig. 2E and F). Overall, the α-diversity of bacteria and fungi in the Bs group slightly outweighed that of the NC group.

**Fig 3:**
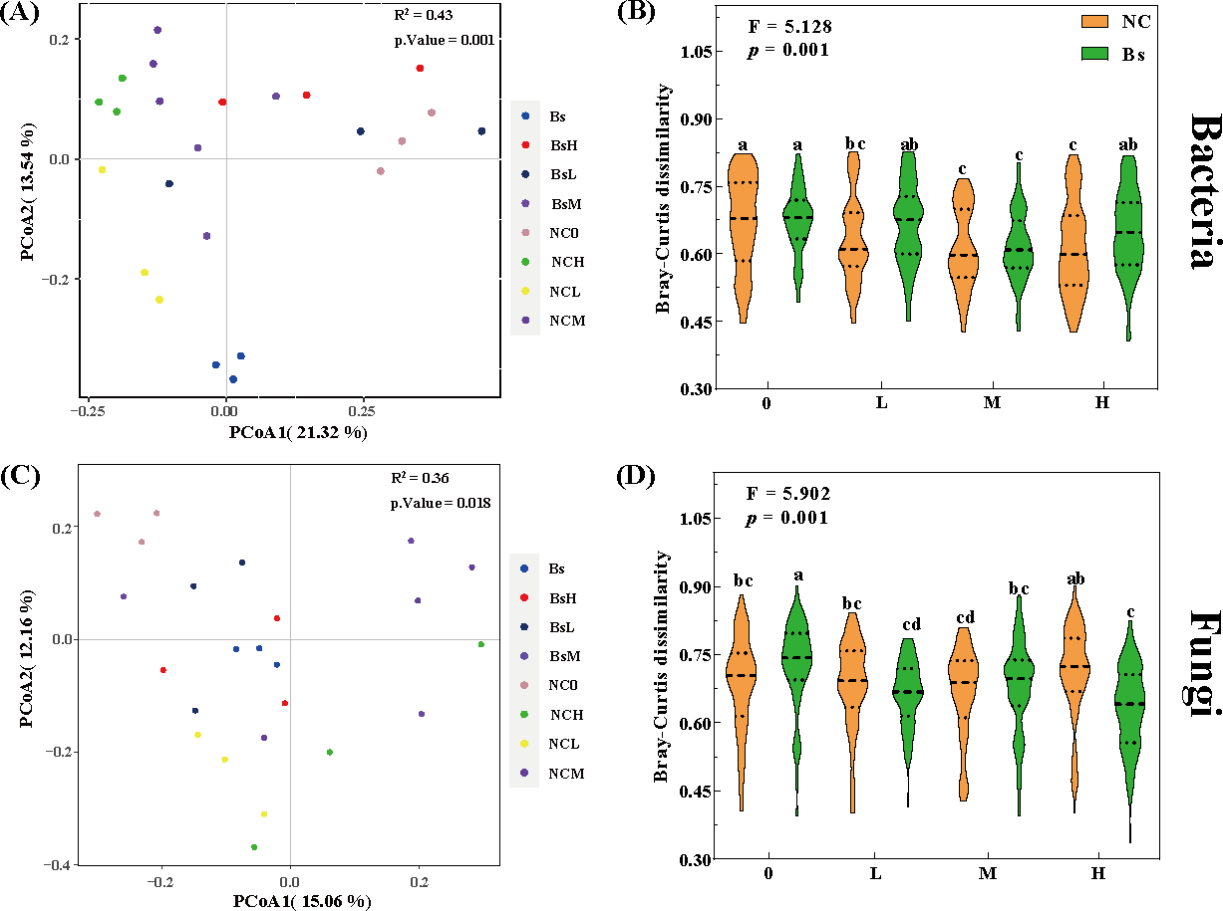
Principal coordinate analysis (PCoA) and pairwise Bray-Curtis dissimilarity of bacterial (**A, B**) and fungal (**C, D**) communities based on Bray-Curtis distances for the eight treatments. The uninoculated Bs group is denoted by yellow, while the inoculated group is represented in green. Different lowercase letters in the same subplot represent significant differences (*P* < 0.05).

The distribution patterns of rhizosphere microbial communities across the eight treatment groups do not exhibit distinct trends. Partial intersections between various groups are observed, yet under varying salt stress levels, clear segregation is evident between pre- and post-inoculation samples with Bs, as validated by ANOSIM (Fig. 3; Table S1). Significantly divergent bacterial and fungal compositions are evident among the treatment groups (Fig. 3A and C, *P* < 0.01 or *P* < 0.05). Similarly, at the bacterial level, BsH exhibits significantly higher abundance than NCH, whereas this trend is reversed at the fungal level, as depicted by the Bray-Curtis dissimilarity algorithm (Fig. 3B and D).

### Soil microbial community compositions

**Fig 4:**
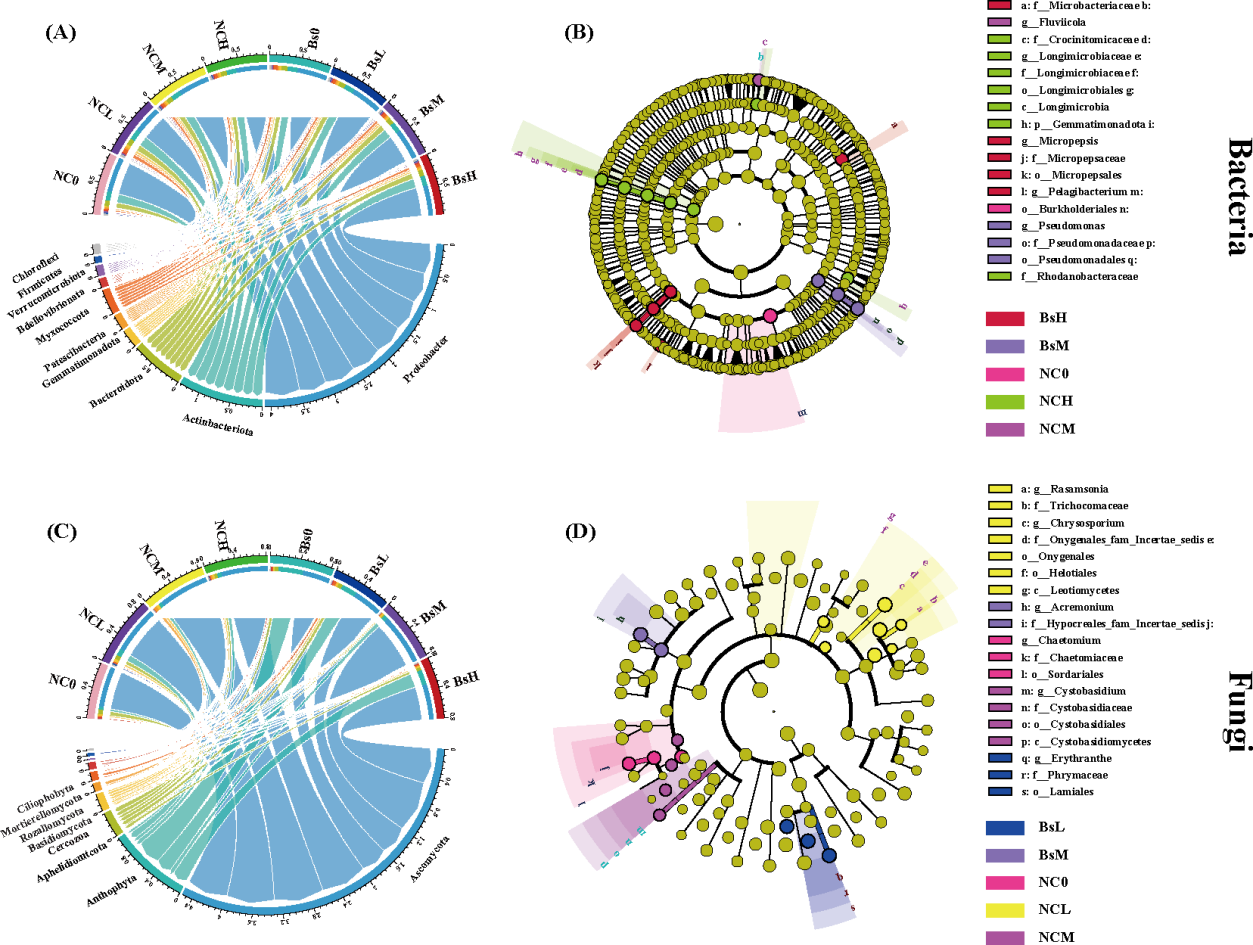
Species composition at the phylum level and distribution of dominant phyla at different taxonomic levels of bacterial communities (**A, B**) and fungal communities (**C, D**) in eight different treatments. The figure depicts the relative abundance of microbial species across treatment groups in a circular layout. Each node represents a microbial species, with the thickness of the edges reflecting the magnitude of relative abundance between microbes, and their spatial arrangement indicating the degree of correlation. The Lefse analysis plots highlight the taxonomic units that exhibit differential abundance across treatment groups at various levels of classification.

The composition of soil microbial communities is depicted in Fig. 4. Within the bacteria category, Proteobacteria (53.11%), Actinobacteriota (16.20%), Bacteroidota (11.13%), Gemmatimonadota (3.44%), Patescibacteria (2.72%), Myxococcota (2.61%), Bdellovibrionota (2.22%), Verrucomicrobiota (2.20%), and Firmicutes (1.73%) were identified as dominant phyla with an average abundance exceeding 1%. Among these, Gemmatimonadota and Bdellovibrionota showed significant differences in abundance across treatment groups (*P* < 0.05). Notably, as stress levels increased, the abundances of Proteobacteria, Actinobacteriota, Bacteroidota, and Firmicutes exhibited a continuous rise, with the Bs group consistently higher than the NC group. Meanwhile, under moderate stress, Gemmatimonadota, Bdellovibrionota, and Verrucomicrobiota displayed the highest abundance (Fig. 4A; Table S2). In the fungal community, Ascomycota (59.42%), Anthophyta (11.49%), Aphelidiomycota (3.12%), Cercozoa (2.79%), Basidiomycota (1.19%), and Chlorophyta (1.14%) were predominant. The relative abundances of Ascomycota and Basidiomycota were significantly influenced by salt stress and inoculant application (*P* < 0.05). As stress levels increased, the abundance of Ascomycota rose, while other fungal phyla exhibited varying degrees of decline. Interestingly, under no-stress and low-stress conditions, Anthophyta’s abundance in the Bs group was significantly higher than that in the NC group (*P* < 0.05) (Fig. 4C; Table S3).

**Fig 5:**
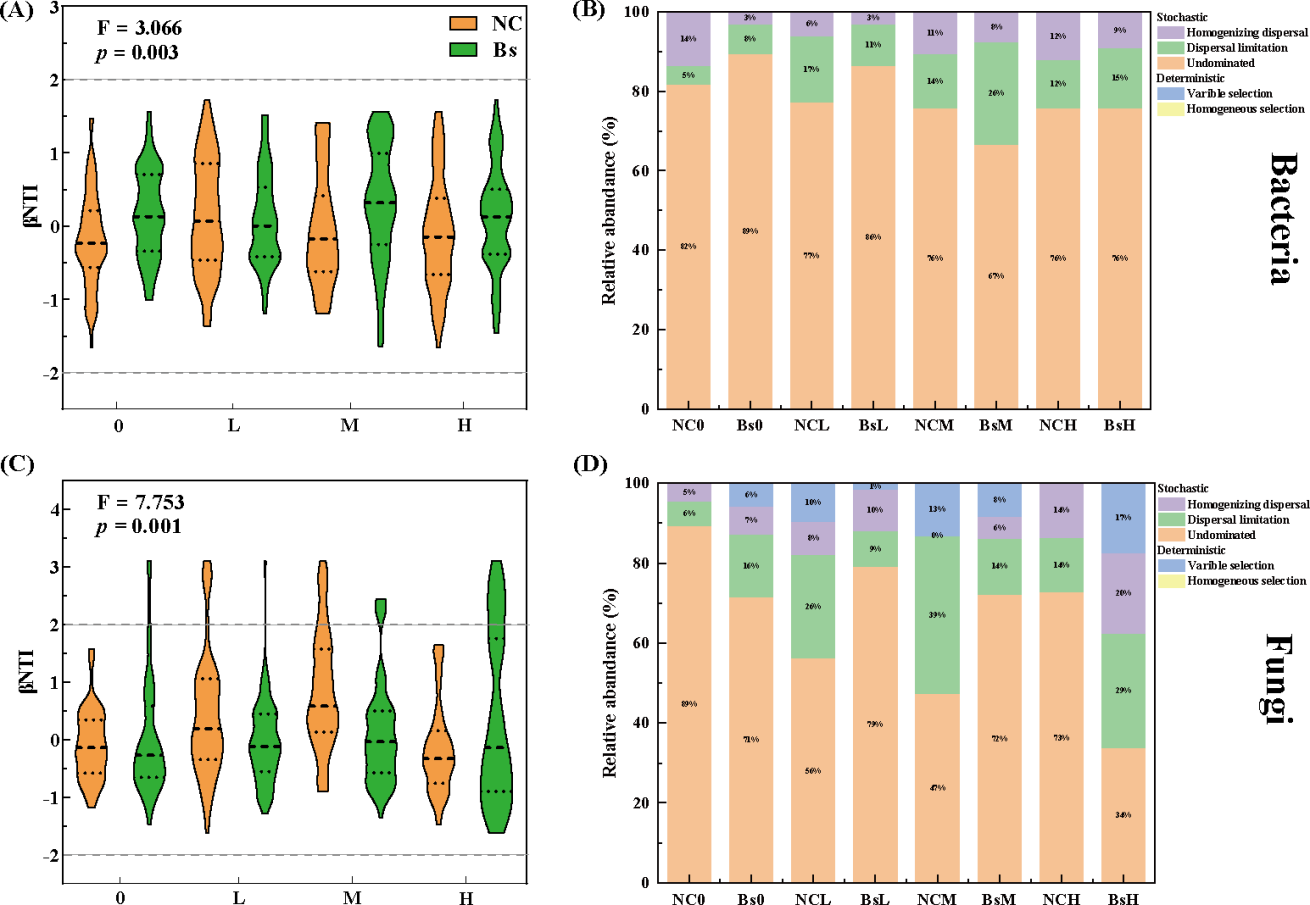
The β-nearest taxon index (βNTI) was employed to conduct stochastic process analyses on bacterial (**A, B**) and fungal (**C, D**) community assemblages across eight treatments. A βNTI value greater than 2 or less than −2 indicates the predominance of deterministic processes, while values between −2 and 2 suggest stochastic processes. The distinct colors depicted in the right panel illustrate the relative percentage contribution of each process to community assemblage.

Additionally, LEfSe analysis, a statistical method for identifying high-dimensional biomarkers and revealing genomic features, was employed (Fig. 4). By setting the LDA threshold above 4, several bacterial taxa highly represented in specific groups were identified, including Microbacteriaceae (family level), Pseudomonadaceae (family level), Burkholderiales (order level), Gemmatimonadota (phylum level), and Fluviicola (genus level). Within the dominant soil fungal taxa, Erythranthe (genus level), Hypocreales (family level), Chaetomium (genus level), Onygenales (order level), and Cystobasidiaceae (family level) were found (Fig. 4B and D).

### Soil microbial community assembly processes

As depicted in Fig. 5, the βNTI score of less than 2 indicates that the assembly dynamics of soil microbial communities are governed by stochastic processes for both bacteria (Fig. 5A) and fungi (Fig. 5C). Specifically, across all groupings, the proportion of stochastic processes for rhizospheric bacteria is 100%. Regarding rhizospheric fungi, aside from NC0 and NCH, which are controlled by 100% stochastic processes, the remaining groups, such as NCL (90%), NCM (87%), Bs0 (94%), Bs0 (99%), BsM (92%), and BsH (83%), exhibit varying degrees of deterministic processes (Fig. 5B and D). These findings indicate that regardless of Bs inoculation, nondominant processes are critical factors influencing the aggregation of rhizospheric bacterial communities, with proportions exceeding 78% (Fig. 5B). However, with increasing salinity stress, dispersal limitation demonstrates a continuous declining trend in the NC group, while in the Bs group, it peaks at medium concentrations. Conversely, among rhizospheric fungi, variable selection begins to manifest in numerous groupings, and with the elevation of stress concentration, its proportion gradually increases in the Bs group, displaying a similar trend of dispersal limitation (Fig. 5D).

### Soil microbial community co-occurrence network

**Fig 6:**
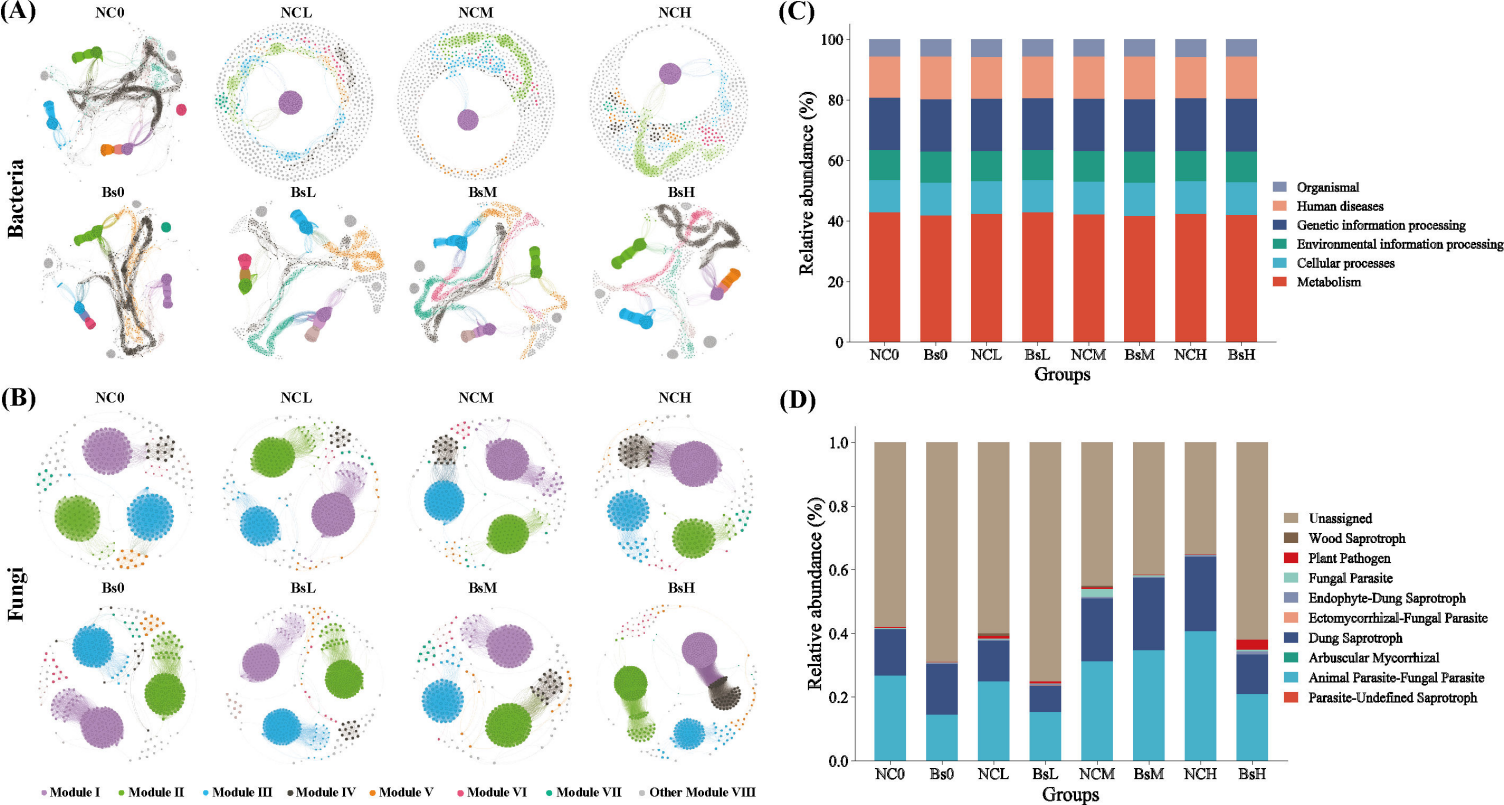
Co-occurrence network and function prediction. Network analyses showing the co-occurrence patterns of bacteria (**A**) and fungi (**B**) for each treatment. (**C**) Histogram of predicted relative abundance of bacterial PICRUSt2 functions. (**D**) Spectral relative abundance of functional groups prediction for fungi. The edges indicate the correlation between the two nodes. A connection represents a strong (*r* > 0.6) and significant (*P* < 0.001) correlation. Different colors represent different modules.

To further elucidate the ecological dynamics of soil microbial communities within the inter-root region of licorice subjected to varying levels of salt stress and subsequent inoculation with strain Bs, the present study has conducted a symbiotic network analysis (Fig. 6A and B), the magnitude and average node connectivity of the bacterial community within the soil notably surpassed those of the fungal community (Table S4). Within the inter-root microbial network, elevations in salt stress concentration fostered enhanced interconnectivity among microorganisms within a certain threshold, yet a diminution was observed at higher concentrations. This suggests that salt stress may facilitate the aggregation of interconnected OTUs within the inter-root microbial network. Intriguingly, both bacteria and fungi exhibited markedly increased edges, nodes, and average weightedness after Bs inoculation and salt stress imposition. This suggests that the role of Bs in augmenting salt tolerance in licorice may also entail the recruitment of additional microorganisms, thereby enhancing microbial diversity—a trend accentuated particularly at moderate to high concentrations. Moreover, the modularity values of bacterial and fungal interactions consistently surpassed 0.4 across all treatments, indicating a modular network structure (Table S4).

### Prediction of soil microbial function

Based on PICRUSt2, functional predictions of bacterial communities were conducted, aligning sequencing data against the KEGG database, which encompassed six identifiable biological metabolic pathways (Fig. 6C). These pathways include Metabolism, Cellular Processes, Environmental Information Processing, Genetic Information Processing, Human Diseases, and Organismal. Notably, “Metabolism” emerged as the predominant functional category, encompassing activities such as carbohydrate metabolism, amino acid metabolism, lipid metabolism, and energy metabolism, with abundances ranging from 41.8% to 42.9%. It was observed that in the un-inoculated “Bs” group, the proportion of metabolic functions was consistently higher than in the “Bs” inoculated group, despite the marginal nature of this difference. Using FUNGuild to predict the functional profiles of rhizospheric fungal communities across different treatment groups (Fig. 6D), a total of 10 functional guilds were identified. Among these, unclassified saprophytes, Animal Parasite-Fungal Parasites, and Dung Saprotrophs were prevalent. Notably, except under moderate stress conditions, Animal Parasite-Fungal Parasite consistently exhibited a higher proportion in the “NC” treatment group compared to the “Bs” group, with this proportion increasing with elevated stress levels. Similar trends were also evident in the case of Dung Saprotrophs.

## DISCUSSION

In nature, salt plays a crucial role in various metabolic pathways and growth processes of plants. The soil environment serves as a vital medium for plant growth and nutrient acquisition, with salt concentration being one of the key environmental factors, especially for plants such as licorice growing in saline-alkali soils. In previous studies, our research team utilized a combination of physiological and multi-omics analyses to confirm the effectiveness of Bs in enhancing salt tolerance in licorice. At the molecular level, we unveiled that the enhancement of salt tolerance is associated with key genes involved in reinforcing the first barrier formed by plant cell walls against salt stress, achieved through enhanced lignin synthesis and strengthened cell wall division and elongation processes (14). However, it is worth considering that upon inoculation with Bs, the inoculant primarily acts on the soil, impacting the soil microenvironment and biodiversity, and thereby indirectly regulating plant growth and development. Previous studies have demonstrated the significant role of soil enzymes in biotransformation, particularly in the decomposition of soil organic matter and nutrient cycling (20). Salt stress can elevate soil salt concentration, thereby increasing the reverse osmotic pressure of soil water, and limiting the water absorption capacity of plant roots. Simultaneously, it can lead to excessive production of reactive oxygen species, resulting in lipid peroxidation of cell membranes and membrane damage. Enzymes such as peroxidase, urease, and sucrase not only degrade organic matter in the soil, enhance plant antioxidant capacity, and reduce the accumulation of reactive oxygen species, but also catalyze urea hydrolysis, providing carbon sources (21). The current study demonstrates that inoculation with Bs aids in restoring soil enzyme activity, which undoubtedly complements the maintenance of nutrients required for plant growth and development to resist salt stress, a result corroborated in numerous studies on abiotic stress.

The sequencing analyses of 16S rRNA and ITS offer insights into extracting valuable information from microbial data and establishing a communication platform between microorganisms and abiotic factors. Researchers summarized the functional roles and mechanisms of microorganisms under non-biological stress conditions, suggesting that specific organisms exhibit distinct gene expression patterns as ultimate response units under specific non-biological stress conditions (22). These patterns further mediate the transmission of molecular and genetic information, the production of secondary metabolites, and interactions involving cell transduction and signal transduction. In this study, it was observed that both fungi and bacteria in samples inoculated with Bs exhibited a higher number of OTUs, with an increase in stress severity promoting OTU abundance. Conversely, non-inoculated samples displayed the opposite trend. This indicates a more complex microbial network in the rhizospheric soil inoculated with Bs, providing essential substances for soil nutrient conversion, thereby offering crucial insights into understanding the mechanism by which Bs enhances salt tolerance in licorice seedlings. Under different salt stress concentrations, Bs exerted varying effects on the diversity, composition, and assembly processes of the rhizobial microbiota. It is worth noting that, to explore the relationship between the dynamic changes in microbial communities and gene expression, as well as secondary metabolites under salt stress after Bs inoculation, we conducted Mantel test analyses by integrating published transcriptome and metabolome data. We found a close correlation between the dynamic changes in bacterial and fungal communities and organic acids in the roots (Fig. S2). Some scholars believe that the secretion of organic acids can alter the diversity and community structure of soil microorganisms, with certain bacteria and fungi exhibiting selective utilization abilities for specific types of organic acids (23). This may lead to the formation of dominant bacterial and fungal communities around the roots. The Bs strain has been shown to metabolize organic acids such as malic acid, citric acid, and succinic acid, collectively supporting the hypothesis that understanding the microbial community’s response to salt stress from a plant metabolism perspective elucidates potential mechanisms for altering community composition during Bs response to salt stress.

At the community level, the abundance of dominant bacterial phyla associated with soil denitrification and organic matter decomposition, such as Proteobacteria and Firmicutes, remained consistently higher in the Bs group than in the NC group under salt stress conditions. This suggests that the rhizospheric soil inoculated with Bs may exhibit stronger denitrification processes (24), facilitating the reduction of excessive nitrate into nitrogen gas and mitigating salt stress by consuming oxygen. In contrast, Gemmatimonadota was significantly lower in the Bs group. This bacterial phylum is known to thrive in extreme environments such as high temperatures, high salinity, and low oxygen (25). LEfSe analysis indicated that the abundance of microbes associated with extracellular polymer production and osmoregulation varied with increasing stress concentration and the presence of Bs inoculation, such as Microbacteriaceae at the family level and Fluviicola at the genus level (Fig. 4). Additionally, Bs inoculation not only influenced bacterial abundance but also had a cascade effect on fungal ecological balance. Surprisingly, a significant decrease was found in Ascomycota and Basidiomycota, which are associated with organic matter decomposition after Bs inoculation. It is implied that external bacteria may engage in ecological niche competition with certain fungi in the soil, balancing microbial communities to enhance plant salt tolerance. This discrepancy may be attributed to environmental and individual differences, with microbial metabolic activity and dispersal potential possibly influencing the sufficiency of deterministic or stochastic processes, as reported by Anderson and Wu (26, 27) (Fig. 5). Co-occurrence networks can be used to visualize complex relationships among soil microbial communities, revealing potential ecological roles of various soil microbial taxa (28). As shown in Fig. 6, regardless of Bs inoculation, soil bacterial community co-occurrence networks were more complex and stable than fungal networks, indicating the crucial role of bacteria in the microecological regulation of soil environments. It was found that under high salt stress, there were consistently more edges, and positive cooperation may aid community resilience in the face of changing environments, as microbial interaction networks can provide a buffer against environmental disturbances (29).

The role of bacteria in the soil provides insights into soil fertility to a certain extent. Employing PICRUSt2 for the prediction of bacterial community function, all treatment groups primarily exhibit metabolic functions, crucial for the conversion of carbohydrates and ammonium nitrogen in the soil to facilitate efficient nutrient utilization by soil bacteria. However, in the short-term treatments, the functional variations resulting from the introduction of Bs did not yield significant differences. Regarding the forecast of fungal function, Dung Saprotrophs play a key role in decomposing organic matter such as plant residues and animal feces, positively influencing plant growth (30). Yet, observations from the present study indicate that the prevalence of this functional fungus gradually expanded with increased stress concentration. Surprisingly, the treatment group inoculated with Bs showed a lower proportion compared to the NC group, contradicting the initial expectations. This discrepancy may be attributed to the high adaptability and competitive stress environment of fungi.

In summary, higher salt stress concentrations appeared to lead to a decline in soil enzyme activities, whereas Bs inoculation aided in improving plant access to nutrients. Microbiome analysis revealed that Bs inoculation increased both the quantity and diversity of bacterial and fungal OTUs, notably enhancing the abundance of Proteobacteria and Firmicutes, thereby enhancing the soil denitrification process. This microbiome study significantly enhances our understanding of how biofungicides mitigate abiotic stresses.

## Data Availability

The data and materials that support the findings of this study are available from the corresponding author upon reasonable request. Sequencing original data have been made available in the Sequence Read Archive (SRA) database (https://submit.ncbi.nlm.nih.gov/subs/sra/) under the accession number PRJNA1105426.

## SUPPLEMENTAL MATERIAL

Figure S1 spectrum.03812-23-s0001.pdf Network analyses showing the co-occurrence patterns of bacteria and fungi.

Figure S2 spectrum.03812-23-s0002.pdf Mantel test to analyze the correlation between differential metabolites and differential genes.

Supplemental tables spectrum.03812-23-s0003.docx Tables S1-S4.

ASM does not own the copyrights to Supplemental Material that may be linked to, or accessed through, an article. The authors have granted ASM a non-exclusive, world-wide license to publish the Supplemental Material files. Please contact the corresponding author directly for reuse.
