# Supplementary material for: Enhanced salt tolerance in Glycyrrhiza uralensis Fisch. via Bacillus subtilis inoculation alters microbial community
Source: Microbiol Spectr. 2024 Aug 27;12(10):e03812-23. doi: 10.1128/spectrum.03812-23 (PMC11448385; doi:10.1128/spectrum.03812-23)

Supplementary Figure S1 Network analyses showing the co-occurrence patterns of bacteria (a) and fungi (b) for each treatment. The size of each node is proportional to the degree of connections. The edges indicate the correlation between two nodes. A connection represents a strong (|r| > 0.6) and significant ($p < 0.001$) correlation..

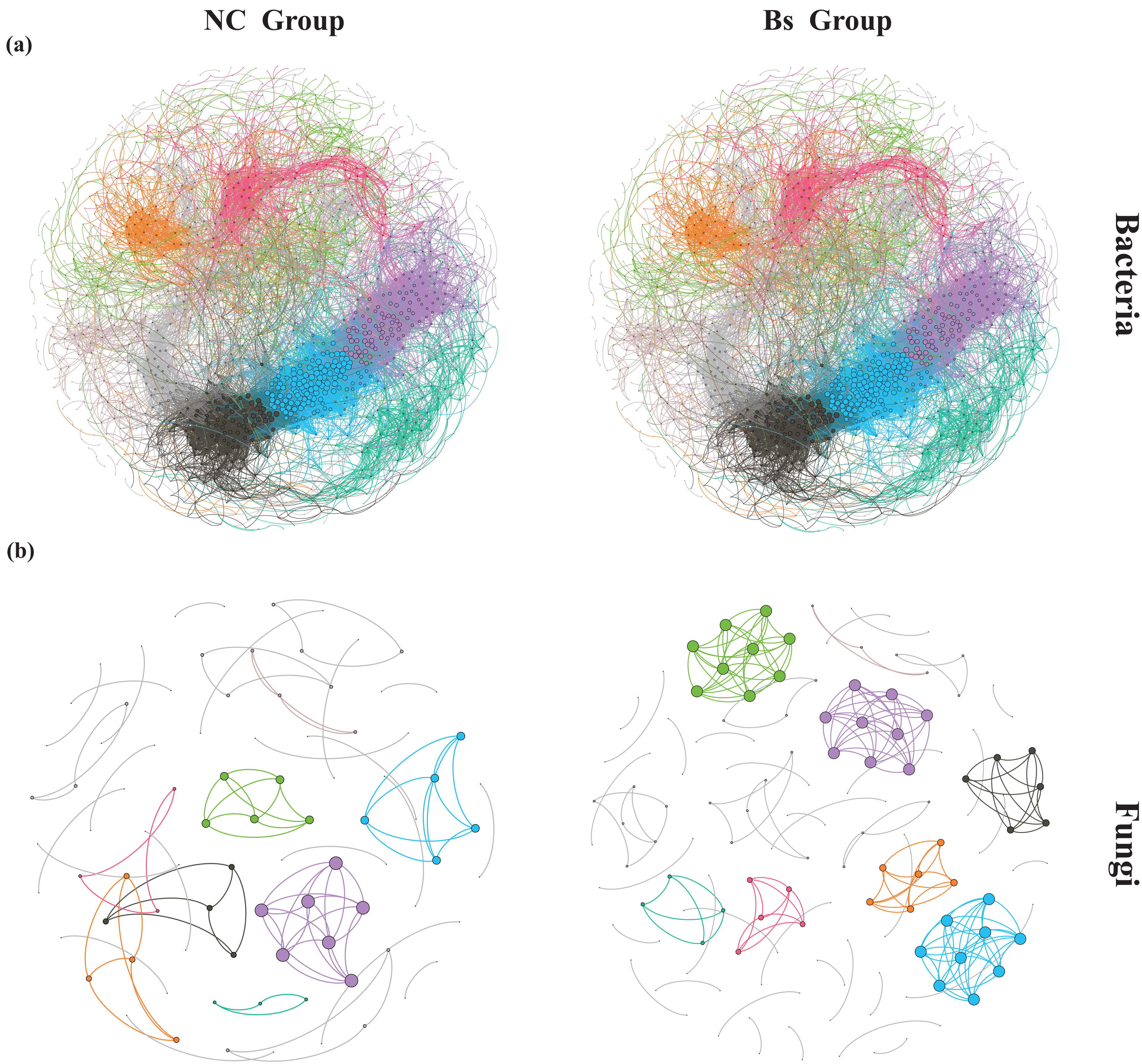

Supplement: Figure S1 — Network analyses showing the co-occurrence patterns of bacteria and fungi. [file spectrum.03812-23-s0001.pdf]
