# Supplementary material for: Enhanced salt tolerance in Glycyrrhiza uralensis Fisch. via Bacillus subtilis inoculation alters microbial community
Source: Microbiol Spectr. 2024 Aug 27;12(10):e03812-23. doi: 10.1128/spectrum.03812-23 (PMC11448385; doi:10.1128/spectrum.03812-23)

Supplementary Figure S2 Mantel test was employed to analyze the correlation between differential metabolites (A) and differential genes (B) in bacterial and fungal communities, respectively. In the visualization, a green color indicates a significant increase in correlation between the two datasets, while yellow denotes a decrease. Red lines signify highly significant correlations between the identified metabolites or genes and bacterial or fungal diversity ($p \leq 0.01$), whereas blue lines represent correlations that are statistically significant ($p \leq 0.05$). The thickness of the lines is positively associated with the magnitude of the correlation coefficients (r).

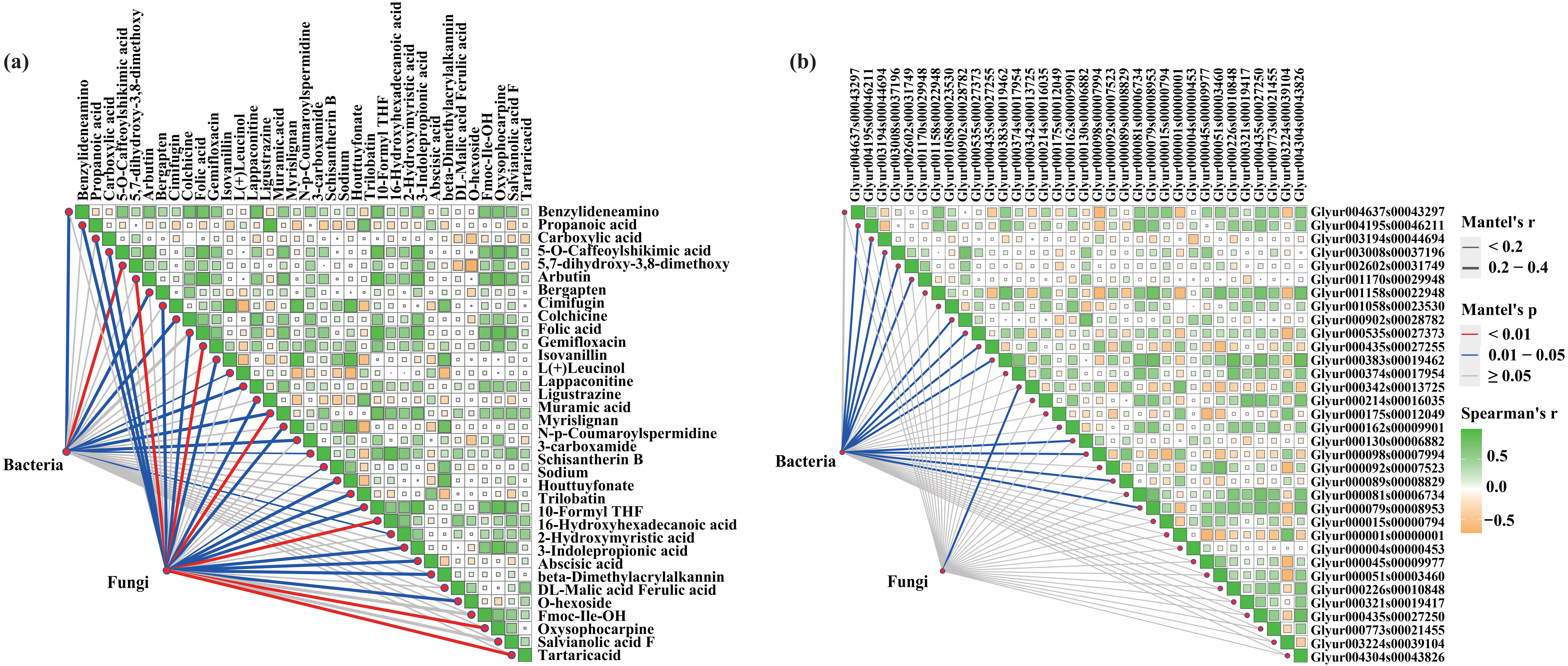

Supplement: Figure S2 — Mantel test to analyze the correlation between differential metabolites and differential genes. [file spectrum.03812-23-s0002.pdf]
