# Supplementary material for: Enhanced salt tolerance in Glycyrrhiza uralensis Fisch. via Bacillus subtilis inoculation alters microbial community
Source: Microbiol Spectr. 2024 Aug 27;12(10):e03812-23. doi: 10.1128/spectrum.03812-23 (PMC11448385; doi:10.1128/spectrum.03812-23)
Supplement: Supplemental tables — Tables S1-S4. [file spectrum.03812-23-s0003.docx]

**Table S1**

Variance partitioning of soil bacterial and fungal community β-diversity (bray-curtis) in analysis of similarities (ANOSIM).

| **Microbes** | **Factor** | **Bray-Curtis** | | | | | |
| --- | --- | --- | --- | --- | --- | --- | --- |
|  |  | ***Df*** | ***SS*** | ***F*** | ***R^2^*** | **Mean square** | **p.Value** |
| Bacteria | Salt and Bs | 7 | 2.0868 | 1.7581 | 0.4348 | 0.2981 | 0.001 |
|  | Residuals | 16 | 2.7131 | - | 0.5652 | 0.1696 | - |
|  | Total | 23 | 4.7999 | - | 1 | - | - |
| Fungi | Salt and Bs | 7 | 2.0003 | 1.3096 | 0.3643 | 0.2858 | 0.018 |
|  | Residuals | 16 | 3.3912 | - | 0.6357 | 0.2182 | - |
|  | Total | 23 | 5.3915 | - | 1 | - | - |

Df: degrees of freedom; SS: sums of squares; F: F test values; R^2^: variation. p.Values are bolded if significant *p* < 0.05.

**Table S2**

Relative abundances (%) of bacterial compositions across taxonomical classification in rhizosphere soils of eight treatments.

| **Phyla** | **NC0** | **Bs0** | **NCL** | **BsL** | **NCM** | **BsM** | **NCH** | **BsH** | **F** | ***p*** |
| --- | --- | --- | --- | --- | --- | --- | --- | --- | --- | --- |
| Proteobacteria | 57.63±9.16a | 55.00±2.52a | 45.90±1.91a | 57.30±9.61a | 50.93±3.60a | 55.06±3.52a | 47.53±1.94a | 55.54±3.20a | 1.411 | 0.268 |
| Actinobacteriota | 12.15±1.32ab | 13.63±4.15ab | 10.80±0.91a | 15.94±4.85ab | 10.84±2.09a | 11.12±1.26b | 13.43±1.62a | 19.96±2.77a | 2.277 | 0.082 |
| Bacteroidota | 9.60±2.35a | 11.22±0.69a | 10.80±0.91a | 10.92±4.08a | 10.84±2.09a | 12.60±0.68a | 5.63±0.94a | 9.59±3.46a | 0.662 | 0.701 |
| **Gemmatimonadota** | 1.89±0.94c | 2.02±0.76c | 4.78±1.38ab | 1.61±0.84c | 3.18±0.24bc | 5.60±0.44a | 5.63±0.94a | 2.85±1.87bc | 5.093 | 0.003 |
| Patescibacteria | 2.46±1.38a | 2.03±0.38a | 3.21±1.27a | 4.10±2.08a | 2.78±1.47a | 2.69±1.14a | 3.30±0.60a | 1.21±0.22a | 1.032 | 0.447 |
| Myxococcota | 2.63±1.59ab | 3.53±2.36ab | 6.67±3.72a | 2.92±3.75ab | 1.32±0.28b | 1.16±0.56b | 2.02±1.01ab | 0.59±0.22b | 1.563 | 0.217 |
| **Bdellovibrionota** | 1.69±0.86b | 1.74±0.49b | 1.74±0.59b | 1.61±0.53b | 3.04±0.41a | 2.96±0.31a | 2.67±0.31ab | 2.17±0.33ab | 2.776 | 0.043 |
| Verrucomicrobiota | 2.07±0.98a | 2.09±0.56a | 1.97±0.70a | 1.09±0.51a | 2.80±1.14a | 2.85±1.49a | 1.73±0.96a | 1.70±0.98a | 0.720 | 0.657 |
| **Firmicutes** | 0.54±0.18ab | 0.73±0.23b | 0.49±0.06b | 1.60±0.88ab | 1.78±0.33ab | 2.50±1.49ab | 1.19±0.17b | 3.65±0.93a | 2.905 | 0.045 |

Values followed by a different letter within the same column are significantly different at *p* < 0.05.

**Table S3**

Relative abundances (%) of fungal compositions across taxonomical classification (Phyla, Class, and Order) in rhizosphere soils of eight treatments.

| **Phyla** | **NC0** | **Bs0** | **NCL** | **BsL** | **NCM** | **BsM** | **NCH** | **BsH** | **F** | ***p*** |
| --- | --- | --- | --- | --- | --- | --- | --- | --- | --- | --- |
| **Ascomycota** | 56.21±22.06abc | 38.21±16.03c | 66.36±15.31abc | 39.65±9.02bc | 69.60±2.51ab | 69.92±13.26ab | 79.48±8.31a | 55.91±6.02abc | 2.592 | 0.049 |
| Anthophyta | 3.57±2.09b | 34.13±25.32a | 9.43±4.14b | 21.49±8.56a | 4.77±2.15b | 5.85±1.62b | 6.41±4.05b | 6.26±2.68b | 2.413 | 0.069 |
| Aphelidiomycota | 6.43±4.79a | 5.14±2.25a | 5.47±5.82a | 5.65±1.20a | 0.18±0.12a | 1.73±1.47a | 0.21±0.19a | 0.61±0.15a | 1.826 | 0.151 |
| Cercozoa | 2.42±0.57ab | 1.95±1.19ab | 3.73±0.33ab | 2.92±1.05ab | 3.78±1.22ab | 1.88±1.19ab | 4.06±0.49a | 1.59±0.91b | 2.174 | 0.094 |
| **Basidiomycota** | 0.55±0.34b | 0.23±0.15b | 0.90±0.32b | 0.82±0.08b | 4.56±1.12a | 0.67±0.41b | 0.72±0.13b | 1.06±0.27b | 17.426 | 0.001 |
| Chlorophyta | 1.16±0.58ab | 0.65±0.23ab | 1.61±1.40ab | 3.87±3.68a | 0.29±0.11b | 0.66±0.44ab | 0.69±0.52ab | 0.24±0.15b | 1.382 | 0.278 |

Values followed by a different letter within the same column are significantly different at *p* < 0.05.

**Table S4**

Topological properties of co-occurrence networks of soil bacterial and fungal communities.

| **Microbes** | **Treatments** | **Nodes** | **Edges** | **Average length** | **Average weighted degree** | **Density** | **Modularity** |
| --- | --- | --- | --- | --- | --- | --- | --- |
| Bacteria | NC0 | 842732 | 3357 | 18.293 | 501.833 | 0.15 | 0.581 |
|  | Bs0 | 698645 | 3532 | 16.027 | 395.468 | 0.112 | 0.673 |
|  | NCL | 87385 | 1135 | 5.07 | 153.979 | 0.136 | 0.42 |
|  | BsL | 546618 | 2787 | 33.228 | 382.042 | 0.141 | 0.6 |
|  | NCM | 153563 | 1385 | 4.994 | 221.746 | 0.16 | 0.033 |
|  | BsM | 522719 | 3075 | 35.94 | 339.850 | 0.111 | 0.674 |
|  | NCH | 124084 | 1281 | 5.26 | 193.721 | 0.151 | 044 |
|  | BsH | 358174 | 2669 | 17.747 | 268.309 | 0.101 | 0.662 |
| Fungi | NC0 | 9159 | 337 | 1.646 | 54.332 | 0.162 | 0.673 |
|  | Bs0 | 23160 | 433 | 1.926 | 114.947 | 0.248 | 0.747 |
|  | NCL | 35729 | 433 | 1.637 | 195.454 | 0.382 | 0.421 |
|  | BsL | 50340 | 507 | 1.646 | 251.344 | 0.392 | 0.697 |
|  | NCM | 13187 | 385 | 1.526 | 68.377 | 0.18 | 0.666 |
|  | BsM | 16177 | 424 | 1.621 | 76.271 | 0.178 | 0.655 |
|  | NCH | 14752 | 391 | 1.527 | 75.428 | 0.193 | 0.529 |
|  | BsH | 42106 | 628 | 1.457 | 134.033 | 0.214 | 0.522 |
